# Reflecting on the current scenario and forecasting the future demand for medical doctors in South Africa up to 2030: towards equal representation of women

**DOI:** 10.1186/s12960-021-00567-2

**Published:** 2021-03-02

**Authors:** Ritika Tiwari, Angelique Wildschut-February, Lungiswa Nkonki, René English, Innocent Karangwa, Usuf Chikte

**Affiliations:** 1 Division of Health Systems and Public Health, Department of Global Health, Faculty of Health and Medical Sciences, Stellenbosch University, Tygerberg Hospital, Francie Van Zijl Dr, Cape Town, 7505 South Africa; 2 Research and Policy, The National Student Financial Aid Scheme, Cape Town, South Africa; 3 Department of Sociology, University of Pretoria, Pretoria, South Africa; 4 Department of Statistics and Population Studies Programme, University of the Western Cape, Cape Town, South Africa

**Keywords:** Gender, Inequity, Health policy, South Africa, 2030, Medical doctors, Medical profession, Human resources for health, Health systems planning

## Abstract

**Background:**

Increasing feminization of medical professions is well-acknowledged. However, this does not always equate to equitable representation of women within medicine, regarding their socio-demographic indicators, regions, sectors and fields of practice. Thus, this paper quantifies the gap in supply of female medical doctors in relation to demand, towards reaching different gender equity scenarios.

**Methods:**

A retrospective review of the Health Professions Council of South Africa’s (HPCSA) database on registered medical doctors (medical practitioners and medical specialists) from 2002 until 2019 was utilized as an indicator of supply. Descriptive statistics were used to summarize data, and inferential statistics (considering a significance level of 0.05) were utilized to determine the association between the number of male and female doctors, disaggregated by demographic variables. We forecasted future gaps of South African male and female doctors up to 2030, based on maintaining the current male-to-female ratio and attaining an equitable ratio of 1:1.

**Results:**

While the ratio of female doctors per 10 000 population has increased between 2000 and 2019, from 1.2 to 3.2, it remains substantially lower than the comparative rate for male doctors per 10 000 population which increased from 3.5 in 2000 to 4.7 in 2019. Men continue to dominate the medical profession in 2019, representing 59.4% (27,579) of medical doctors registered with the HPCSA with females representing 40.6% (18,841), resulting in a male-to-female ratio of 1:0.7. Female doctors from the Black population group have constantly grown in the medical workforce from 4.4% (2000), to 12.5% (2019). There would be a deficit of 2242 female doctors by 2030 to achieve a 1:1 ratio between male and female medical doctors. An independent-samples t-test revealed that there was a significant difference in the number of male and female doctors. The Kruskal–Wallis test indicated that there was a sustained significant difference in terms of the number of male and female doctors by population groups and geographical distribution.

**Conclusions:**

Based on the investigation, we propose that HRH planning incorporate forecasting methodologies towards reaching gender equity targets to inform planning for production of healthcare workers.

## Background

The history of medicine in South Africa is one of stark and intractable inequality. This is highlighted in the disparities with regards to the provision of health-care services for its citizens, the wide-ranging discrepancies in resource allocation between public/private and urban/rural sectors, as well as the skewed gender and racialized representation of healthcare providers. The profession is shaped distinctively by the segregationist policies of the apartheid system in South Africa, as well as by the overwhelming “patriarchal structures of a largely colonial society” [1]. Recent research points to systemic gender discrimination and inequalities in pre-service and in-service health education and employment [4–6]. Gender discrimination and inequality in the health workforce have received scant attention by HRH leaders and researchers which impacts on the delivery of optimal and equitable healthcare [7]. South Africa ranks as one of the most unequal countries in the world [8, 9]. This labour market inequality is mirrored in the statistics for post-school education and training participation and attainment of educational qualifications. Income inequality in South Africa is pervasive, intractable and affecting every area of life, with those classified as Black trapped in the lower ends of the inequality scale.

Since 1994, attempts at health reforms have focused on the creation of a more equitable and less fragmented health system with a simpler, more efficient regulatory framework. One of the first major policy imperatives for change was contained in the 1994 *National Health Plan *[2], which was considerably developed in the *White Paper for the Transformation of the Health System in South Africa *[3]. This document is the driving force for rectifying the racialized, gender and regional disparities in the South African health system.

The most important interventions the Department of Health instituted related to the:

- *Streamlining of regulatory systems* For example, the professional regulatory bodies which were previously characterized by fragmentation were consolidated into the South African Nursing Council (SANC) and Health Professions Council of South Africa.
- *Addressing of regional imbalances* The abolition of segregated health departments was designed to decrease wastage of limited financial resources, improve poor infrastructure, and attend to the lack of facilities and address instances of poor equipment and a shortage of personnel in the former homelands.
- *Promotion of equality in terms of population groups and access to training* Various measures have been put in place to upgrade and enhance growth in the output of candidates classified as Black, who were previously denied equal access to education and training, and
- *Shifting of focus to primary/community-based health care* as well as the tenets of holistic care, with emphasis on certain diseases such as TB and HIV and AIDS.[4]

The systems of medical professionalization, colonialism, patriarchy, and politics are deeply intertwined. While considerable improvements have been realized since 1994, assessment of inequalities in the system is essential as the salience of the effect of these policies still defines representation in medicine as well as the distribution and dispensation of health care.^1^ The first person of African descent joined the South African health workforce only in 1883 [5]. After more than half a century, the first female doctor of African descent registered in the South African workforce [6].

This study focusses on fair representation of women in medicine, as a first step towards understanding the various forms of inequality in the profession. The increasing feminization of medical profession is well acknowledged across countries. However, in South Africa, little is known about the extent of disparity in female representation in medicine across regions, sectors, population groups, areas of specialization and leadership positions—to inform health workforce planning and policy towards a more gender equitable^2^ workforce. By forecasting the need for medical practitioners and considering equally the need for fair representation of women in the medical workforce, this study provides a view of gaps towards reaching gender equity targets, to inform policy-makers towards addressing the inequalities in future human resources for health (HRH) in South Africa. Workforce planning can be a powerful tool to achieve equitable levels and mixes of HRH availability, to deliver required services to a target population [7].

We forecasted the requirement for medical doctors within South Africa up to 2030 using a manpower-to-population ratio method with a gender equity lens. While estimations of the gap between the supply and demand of selected healthcare professions have been conducted in the country, to our knowledge, this HRH planning and forecasting approach is the first-of-its-kind in that it has been conceptualized with gender equity as a focus. We hope this kind of gender equity-focused HRH planning approach will encourage government, funding agencies and foundations from similar middle-income countries to conduct research programmes examining the integration of forecasting models towards gender equity in workforce planning.

## Methods

The World Health Organization (WHO) classifies health workers into five groups: health professionals, health associate professionals, personal care workers in health services, health management and support personnel, and ‘other’ health service providers not classified elsewhere (which includes medical students, hospital volunteers and members of the armed services). The health professionals further include several categories such as generalist medical practitioners and specialist medical practitioners [10]. Medical doctors (specialists and generalist medical practitioners) serve a key role in health-care provision in health systems [8]. In this study we focus on medical doctors (Appendix).

### Retrospective record review

This was a retrospective record-based review of the Health Professions Council of South Africa (HPCSA) database from 2000 until 2019. The database was accessed by the Department of Global Health, Stellenbosch University through a written request to the HPCSA. This database included data on all medical doctors registered as ‘Medical Practitioners’ (including medical specialists) disaggregated by age, gender, population group, 3 geographical location and category of practice. Data were entered on a Microsoft Excel spreadsheet and analysed using the Statistical Package for the Social Sciences (SPSS version 22.0) [9] and RStudio [10]. Summary statistics and graphical representations were used to describe data. Inferential statistics, using the t-test and Kruskal–Wallis tests, were employed to determine the association between the number of male and female doctors and demographic variables such as age, population group and geographical distribution. Ethical approval and a request for waiver of informed consent for this retrospective study was obtained from the Stellenbosch University Health Research Ethics Committee (HREC Reference No: X20/03/014).

### Gendered analysis of growth in the number of doctors and forecasting up to 2030

The paper describes and analyses the quantitative growth of doctors in the period 2000–2019, according to different variables (gender, location, age). In addition, it considers this growth to forecast supply deficits based on different gender equity scenarios.

Furthermore, we forecasted the future gap for male and female doctors in South Africa up to 2030 (as per the status quo male-to-female ratio and to strike a 1:1 male-to-female ratio). For forecasting, two data points were used in a mathematically simulated excel model: (1) historical trends in the registration of medical doctors (HPCSA record-based review (2000–2019)) to project future trends in the supply of medical doctors (2020–2030) and (2) supply forecasts and trends in population growth, to estimate the match between supply and demand for medical doctors based on two gender equity-based scenarios—status quo and gender equity target of 1:1.

- *Scenario 1* Projects the difference between the demand and supply of female medical doctors in 2030 based on the same rate of production as was the case between 2009 and 2019.
- *Scenario 2* Projects the difference between the demand and supply of female medical doctors in 2030 based on an increased rate of production of female medical doctors from 2020—2030.

These scenarios were developed to understand the current supply trends and estimate the future needs to promote gender equity in the medical workforce and strengthen the recruitment, retention, and upward mobility of women in the medical profession. The basic model for forecasting was adopted from a similar study, which forecasted HRH requirements for the future [11]. HPCSA registrations were used to obtain historical trends of the supply in medical doctors. This addresses the limitations associated with the use of graduation rates as supply^4^ indicator. Based on the historical trends gathered from the HPCSA database from 2000 till 2019 (the last 20 years), the supply of male and female doctors was forecasted using an exponential smoothing technique (time series forecasting method for univariate data that can be extended to support data with a systematic trend or seasonal component) [12]. In this technique, recent observations are weighted more heavily than earlier observations [13]. Thus, the trend in the supply of male and female doctors was used to forecast and estimate the gap in two scenarios based on their density per 10,000 population. The projected total population for South Africa for 2020–2030 was obtained from the Thembisa model [14].

## Results

### Gendered analysis of growth in the number of doctors

**Fig. 1:**
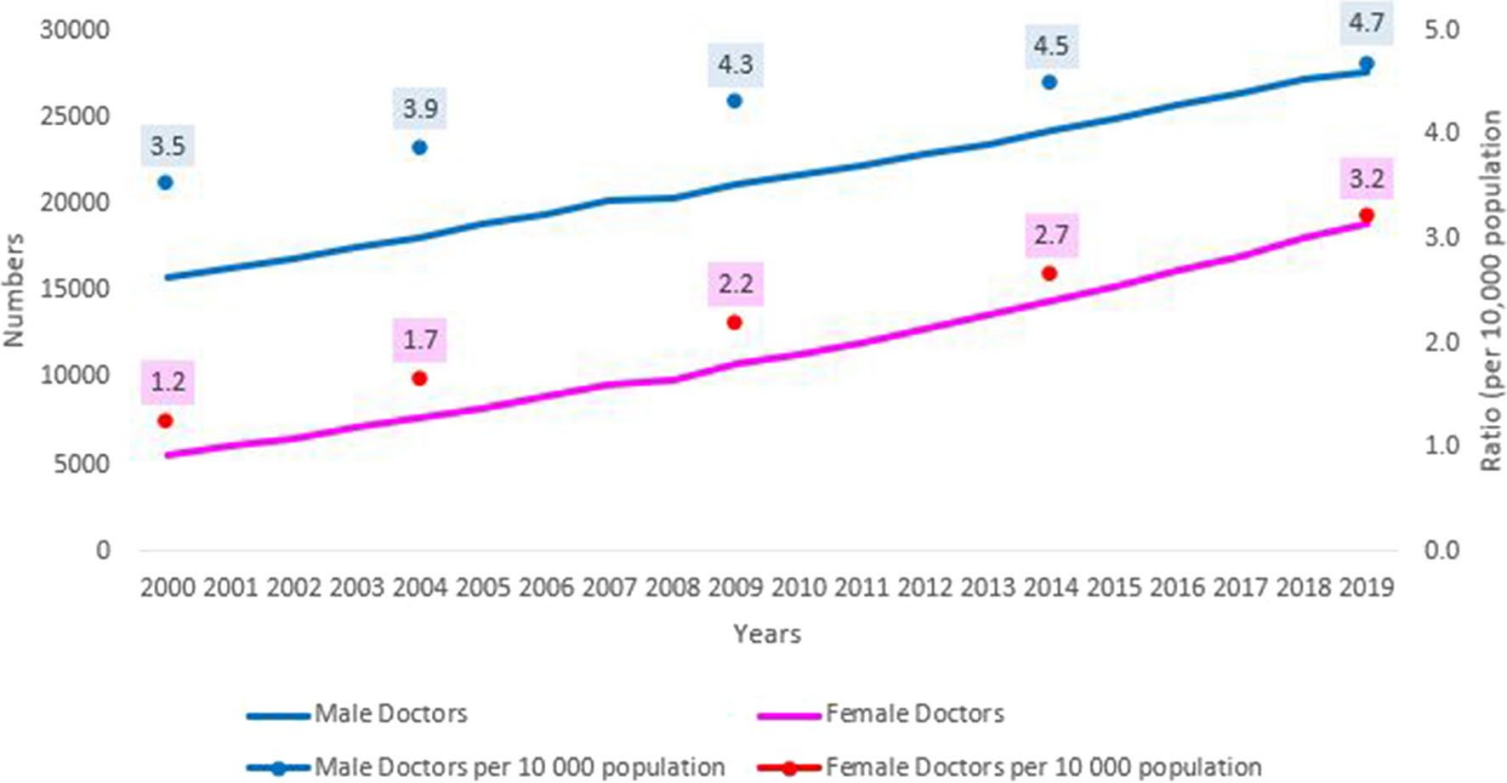
Number of doctors registered from 2000 to 2019 and doctors:10,000 population ratios. This figure represents the number of doctors per 10,000 population from 2000 to 2019

The number of male doctors has increased by 1.7 times from 2000 (*N* = 15,781) to 2019 (*N* = 27,579) with an average annual increase of 3%. The number of female doctors has increased 3.3 times from 5,597 (in 2002) to 18,841 (in 2019), with the average annual increase of 6.6%. While the growth in female doctors has increased, this has been from a low base. As shown in Fig. 1, both the SA population and number of doctors have increased over the 20-years period. Despite this, the gap between males and females has widened over time and there is a significant difference in the average number of male (mean = 21,538, sd = 3695) and female (11,509, sd = 4131) doctors (*p*-value = 0.000 < 0.05).). Compared with a 31.1% increase in the South African population, the ratio of male doctors per 10,000 population in SA also increased from 3.52 in 2000 to 4.69 in 2019. Whereas female doctors per 10,000 population increased from 1.25 in 2000 to 3.21 in 2019. It is noteworthy that female doctors in 2019 have a lower density per 10,000 population compared to male doctors in 2000.

### Geographical distribution by province

**Fig. 2:**
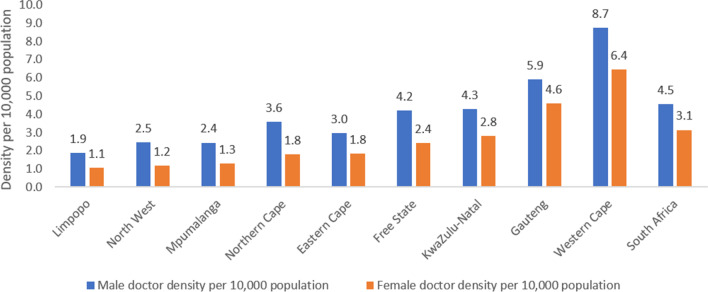
Density per 10,000 population of male and female doctors in 2019. *This figure represents the density of male vs female doctors per 10,000 population at provincial and national level in 2019*

**Fig. 3:**
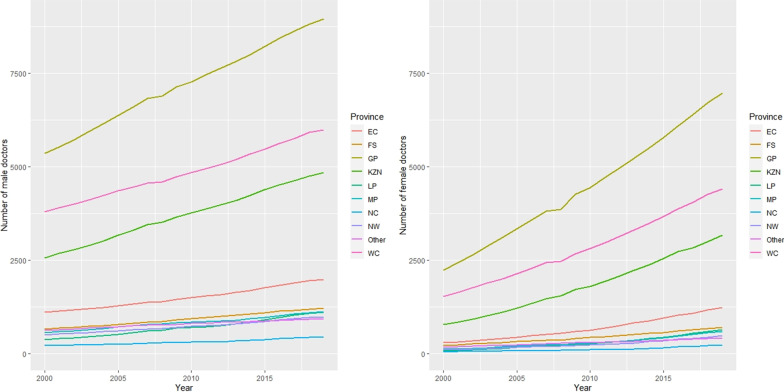
Distribution of male and female doctors per year by provinces

Majority of male doctors are located in the more densely populated and urbanized provinces of Gauteng (*n* = 8,941, density-5.89), Western Cape (*n* = 5984, density-8.74) and KwaZulu Natal (*n* = 4,841, density-4.29). Similarly, majority of female doctors are also located in these provinces, i.e. Gauteng (*n* = 6,972, density-4.59), Western Cape (*n* = 4,405, density-6.44) and KwaZulu Natal (*n* = 3,173, density- 2.81) (Table 1, Fig. 2). The lowest density of male and female doctors occurs in Limpopo followed by Mpumalanga for male doctors and North West for female doctors. The gap between the number of male and female doctors in different provinces has widened over time and there is a significant difference between the median number of doctors across provinces (*p*-value = 0.000 < 0.05) (Fig. 3).

**Table 1:** Geographical distribution of male and female doctors in 2019

	**Category**	**Male doctor**		**Female doctor**		**Population %**		
		**Number**	**Density per 10,000 population**	**Number**	**Density per 10,000 population**			
						**Male population (%) within province **[15]	**Female population (%) within province **[15]	**% of total SA population**
1	Gauteng	8941	5.89	6972	4.59	50.1	49.9	25.8
2	KwaZulu-Natal	4841	4.29	3173	2.81	47.7	52.3	19.2
3	Mpumalanga	1120	2.44	597	1.30	49.3	50.7	7.8
4	Western Cape	5984	8.74	4405	6.44	49.3	50.7	11.6
5	Limpopo	1115	1.86	641	1.07	47.3	52.7	10.2
6	Eastern Cape	1990	2.96	1234	1.84	47.1	52.9	11.4
7	North West	989	2.46	479	1.19	50.7	49.3	6.9
8	Free State	1213	4.20	699	2.42	48.2	51.8	4.9
9	Northern Cape	452	3.58	226	1.79	49.5	50.5	2.2
	Total	26,645	4.53	18,426	3.14	48.8	51.2	100

Excluding from the total workforce of 46,420, 614 doctors indicated as located outside of South Africa and 735 where location wad indicated as unknown

Figure 3 shows that the number of male and female doctors increased over time and varied by population group. A Kruskal–Wallis test revealed a statistically significant difference in the number of male and female doctors across the different population groups (*p*-value =  < 0.001). Western Cape (WC), Gauteng (GAU), Northern Cape (NC), KwaZulu-Natal (KZN), Free State (FS), Mpumalanga (MP), Eastern Cape (EC), North West (NW), Limpopo (LMP).

Across the provinces, male:female medical doctor ratio ranged between 1:0.5 (Mpumalanga, Northern Cape, North West) and 1:0.8 (Gauteng), whereas the population of South Africa has an almost 1:1 male and female ratio.

### Age distribution

**Fig. 4:**
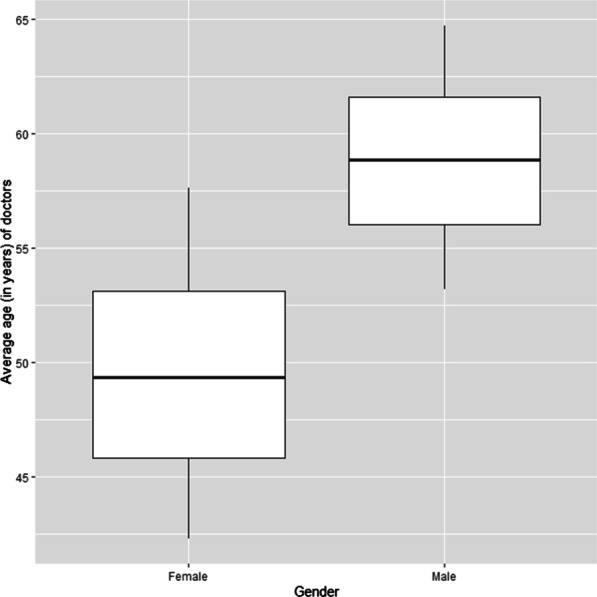
Association between average age and the number of male and female doctors (2019) (*N* = 46,420). This *indicates that there is a negative correlation between the number of male and female doctors and their average age (r* = *-0.99 for both male and female doctors). That is, the number of male and female doctors decreases with the average age*

**Fig. 5:**
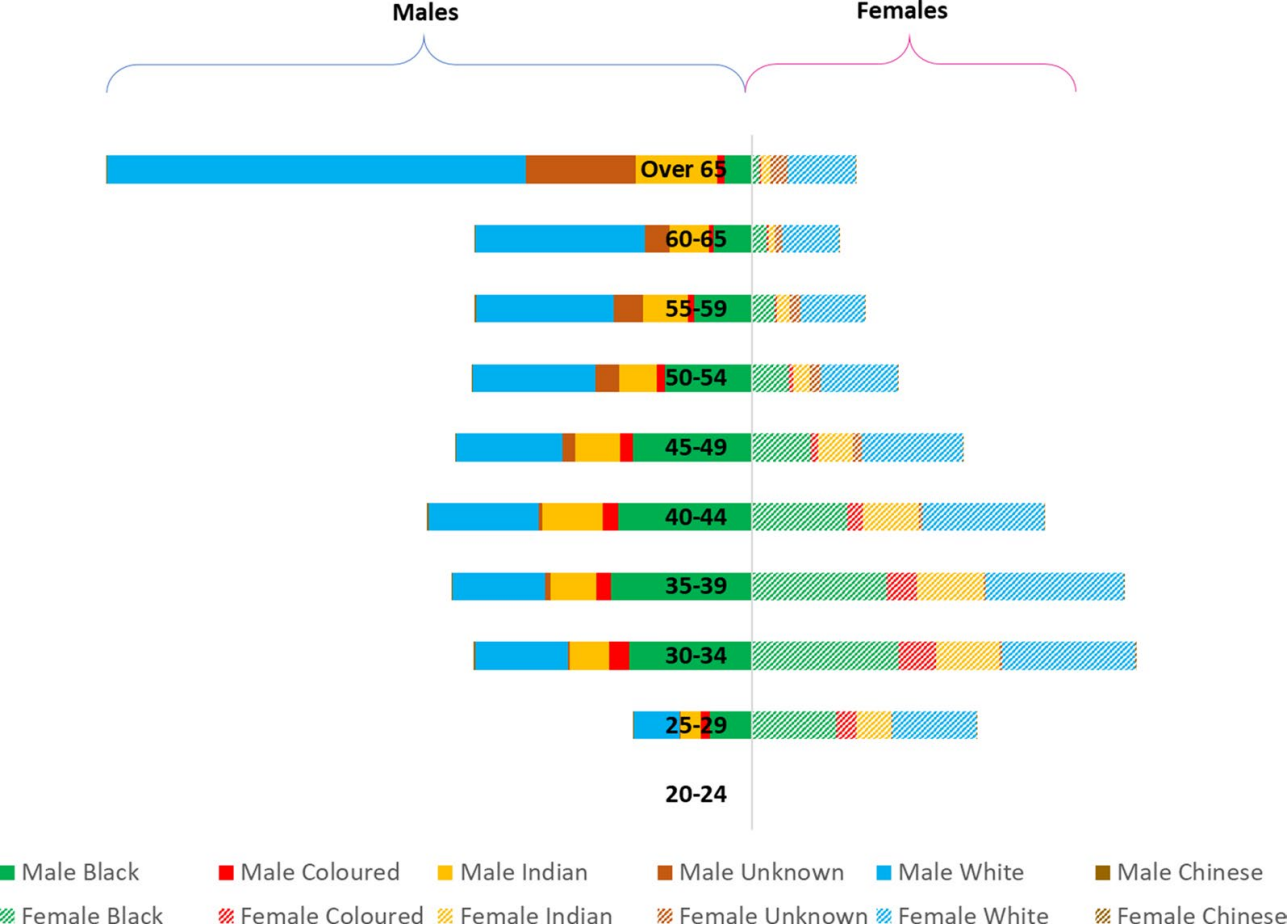
Breakdown of registered doctors by population group, age and sex (South Africa—2019) (*N* = 46,420). *This figure represents the split of registered doctors by population groups, age and sex in 2019*

Excluding the over 65 years category (as per the retirement age in South Africa) [16], the majority of registered male doctors (21.9%) are between the ages 35 and 44 years; whereas, majority of the registered female doctors (38.8%) are between the ages 30 and 39 years.(Fig. 4). As indicated in Fig. 5, there were more male doctors (mean = 59 years, sd = 4 years) than female doctors (mean = 50 years, sd = 5 years). The independent-samples t-test indicated that there was a statistically significant difference in the average age of male and female doctors (p-value).

### Demographic trends by population group, age and sex

Tracking demographic variables of doctors by age, population group and sex (Fig. 5) shows that in 2019 the doctor workforce predominantly comprised males and falling in the population group classified as White across all age groups. The number of doctors decreases with increasing age across all population groups except for the lowest age cohort. Interestingly, females are mostly younger and categorized as White. These trends mimic the trends in enrolment and graduation and universities in South Africa, where females classified as Black and Coloured are poorly represented in comparison to their proportional representation in the populace.[17].

For male doctors classified as White, the majority (43.4%) fall into the age group of 40–59 years; whereas majority (58.2%) of female doctors categorized as belonging to the White population group are in the 30–50 years age group. In the Black population group, the majority (67%) of male doctors belong to the 30–50 years’ age group, whereas 76.6% of female doctors belong to the 25–44 years age group. This reflects the increased entry of young women categorized as Black into the field of medicine, which is a positive sign for a society like South Africa—where ‘race’ is associated strongly with socioeconomic status and consequently, opportunities.[18].

**Fig. 6:**
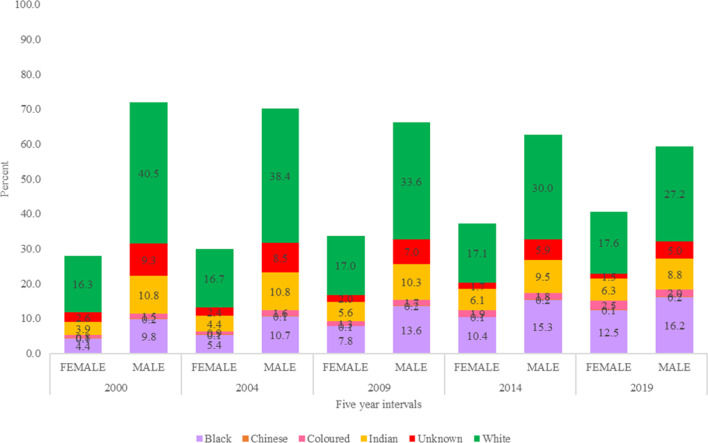
Medical doctors in South Africa, disaggregated by population group (2000–2019). *This figure represents the split of registered doctors in South Africa by sex and population groups in 2000, 2004, 2009, 2014 and 2019*

**Fig. 7:**
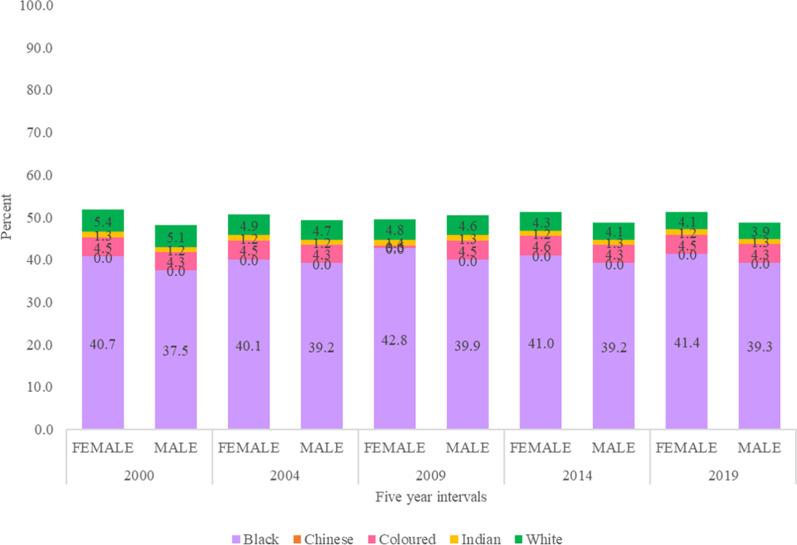
Population of South Africa, disaggregated by sex (2000–2019). *This figure represents the split of population of South Africa by sex and population groups in 2000, 2004, 2009, 2014 and 2019*

**Fig. 8:**
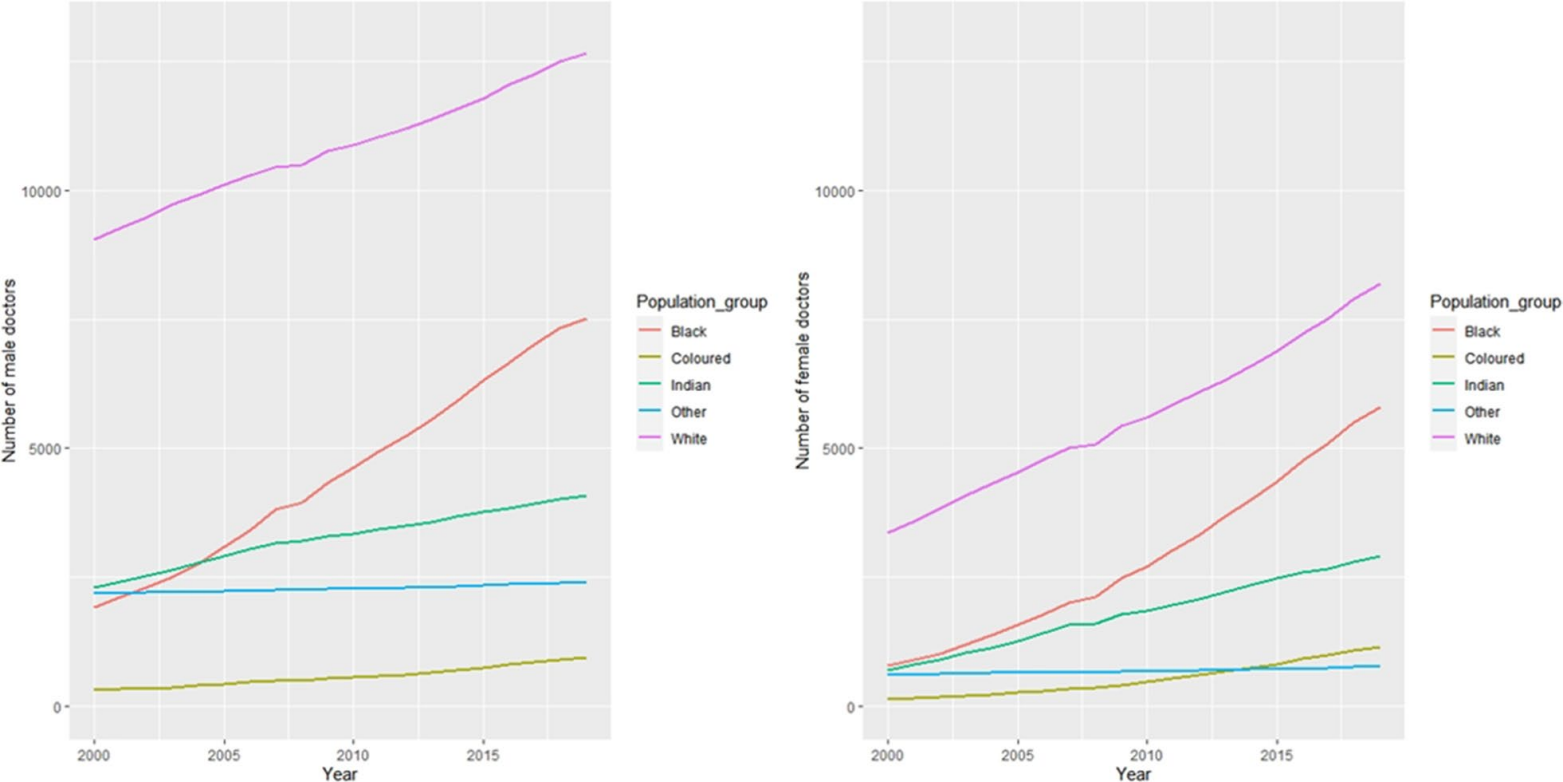
Distribution of male and female doctors per year by population groups. *This figure shows that the number of male and female doctors increased over time and varied by population group. A Kruskal–Wallis test revealed a statistically significant difference in the number of male and female doctors across the different population groups (p-value* =  < *0.001)*

If we analyse the HRH trends as per HPCSA registrations (Fig. 6) for five data points at equal intervals of four years (i.e. 2000, 2004, 2009, 2014 and 2019), then it shows the increase in the proportion of female doctors compared to male doctors from 28.1% (2000), 29.9% (2004), 33.8% (2009), 37.3% (2014) and 40.6% (2019). The proportional representation of women are consistently and considerably lower as compared to the proportional representation of women in South Africa’s population (Fig. 7) (51.9% (2000), 50.7% (2004), 49.6% (2009), 51.2% (2014) and 51.2% (2019)). Additionally, female doctors belonging to the Black population group have constantly grown in the medical workforce from 4.4% (2000), 5.4% (2004), 7.8% (2009), 10.4% (2014) and 12.5% (2019); whereas, Black women have constituted biggest proportion of the South African population over the years, i.e. 40.7% (2000), 40.1% (2004), 42.8% (2009), 41.0% (2014) and 41.4% (2019). The gap between numbers of male and female doctors in different population groups has widened over time and there is a significant difference in the median number of male and female doctors across the population groups (p-value = 0.000 < 0.05) (Fig. 8).

### Composite workforce forecasting

As per the HPCSA database, in the year 2019 there were 27,579 male and 18,841 female doctors registered as ‘Medical Practitioners’. Of these doctors it was assumed that 85% would be active and working in South Africa in 2019. As per a report released by Stats SA in 2015 just under 85% of the South African labour force worked over 40 h a week [19, 20]. Furthermore, on the basis of historical trends as gathered from the HPCSA database for the last 20 years (2000 till 2019), the future supply for male and female doctors was forecasted (Table 2).

**Table 2:** Forecasted annual supply of doctors from 2020 till 2030*

Year	Male	Female
2020	662	965
2021	667	990
2022	672	1014
2023	677	1039
2024	682	1063
2025	688	1088
2026	693	1112
2027	698	1137
2028	703	1161
2029	709	1186
2030	714	1210

*Using exponential smoothing technique

On the basis of active workforce numbers and forecasted supply—net doctors in the South African health workforce were calculated, i.e.

$${\text{Net doctors }}\left( {{2}0{2}0} \right) \, = { 85}\% {\text{ of total registered doctors }}\left( {{2}0{19}} \right) \, + {\text{ forecasted supply for 2}}0{19}{\text{.}}$$

In the first scenario, to maintain the status quo of density of doctors per 10,000 population (i.e. 4.69:3.21) is 1 male vs 0.7 female doctors—the need (for male and female doctors) was estimated for the growing population estimates (up to 2030). In the second scenario, to strike an equity target of 1:1 among the densities of male vs female doctors—since the population of South Africa reflects similar gender proportion of 1:1 (male:female population)—the following need-based scenarios were created (Table 3).

**Table 3:** Equity-based scenarios maintaining status quo of male and female doctors and striking an equity target of 1:1—up to 2030

Scenario	Equity-based scenarios	Doctors per 10,000 population
I	Status quo—male and female doctors	4.69:3.21 = 1:0.7
II	Equity—male and female doctors	4.69:4.69 = 1:1

**Fig. 9:**
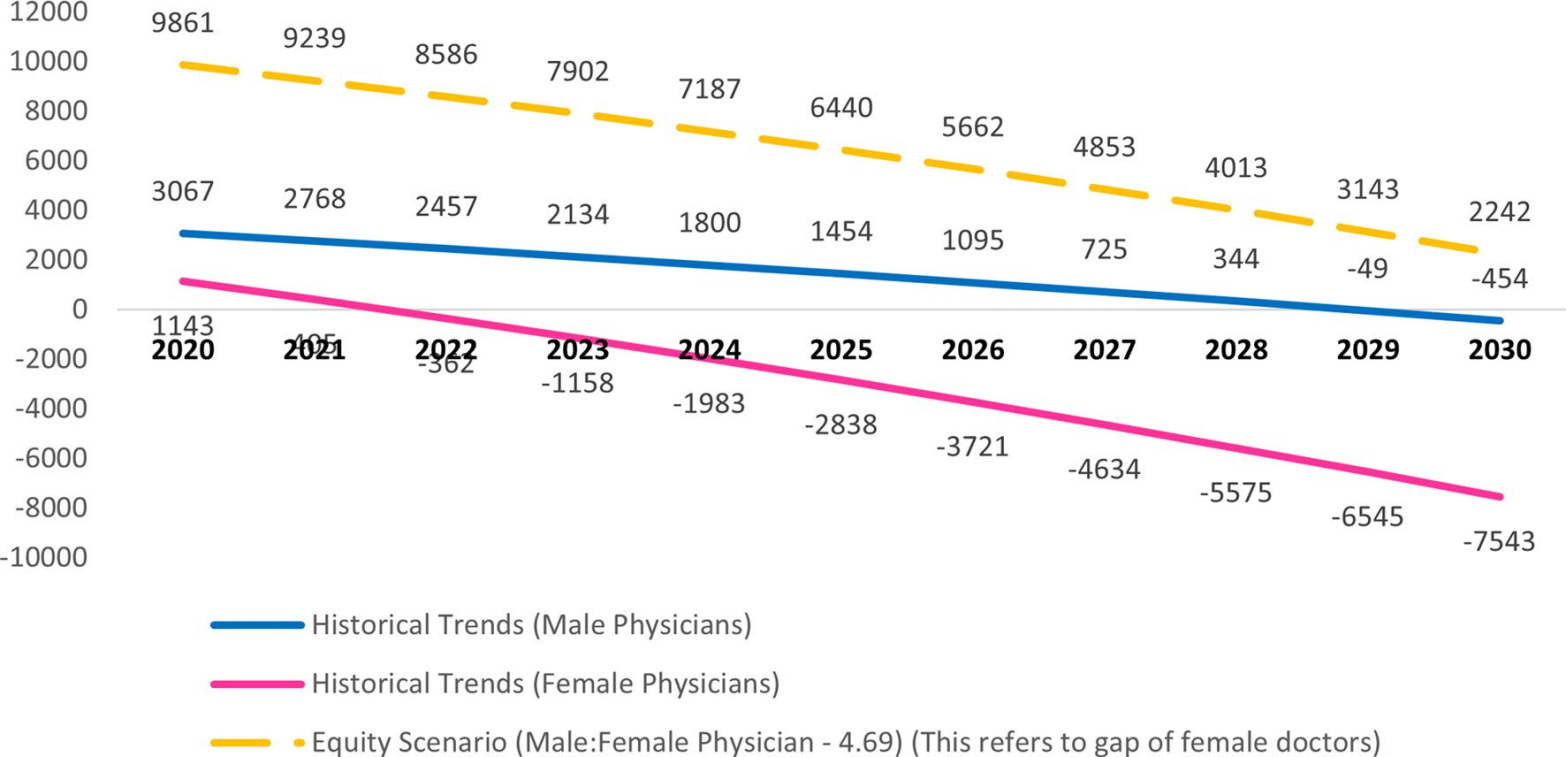
Forecasted gap in the number of doctors, according to level of policy intervention. This figure shows forecasted gap in number of doctors by sex from 2020 till 2030

For calculating the gap in number of doctors, the number of net doctors in the workforce was deducted from the estimated need in South African society. The result of this mathematical simulation produces a forecast gap for male and female doctors in the two scenarios (Fig. 9):

$${\text{Additional need }}\left( {{\text{gap}}} \right) \, = {\text{ net doctors in workforce }} - {\text{ estimated need}}{.}$$

Also, it was assumed that the net workforce of the previous year (n-1) will be the active and working workforce for the next year (n). Since the analysis was drawn not to provide granular, detailed forecasts but rather to give an overarching view of possible directions of change, in order to inform policy-making, reasons for attrition, such as migration, death, change of profession and retirement were not included.

Thus, it was estimated that if the status quo is maintained for male doctors (namely, 4.69 per 10,000 population), then by 2030 there will be the density of male doctors will increase up to 4.76 per 10,000 population. If the status quo is maintained in the production of female doctors (namely, 3.21 per 10,000 population), then by 2030 the density of female doctors will increase up to 4.35 per 10,000 population. However, if we try to strike equity between male and female doctors by target density of 4.69 female doctors per 10,000 population to achieve a 1:1 ratio for male versus female doctors then there would be a need for 2,242 female doctors by 2030.

## Discussion

Improving the equality in representation of women, and particularly women of colour, in education, training and employment, remains a compelling goal. Despite continued policy commitment, there is a persistent under-representation and inequity in treatment and salaries of women, more so with women of colour in different locations and fields of specialization. Within the South African context, the intersectional nature of inequality^5^ thus cannot be ignored.

In the United States of America (USA) in 2015, more than one third (34%) of the active doctor workforce was female [21] and an estimated 46% of all doctors-in-training and more than half of all medical students were women. However in the United Kingdom a pronounced gender gap among specialist doctors was observed with 1:0.5 qualified male vs female specialists on the register [21]. Although on the general practitioner (GP) register, women outnumbered men with 1:1.2 ratio for male vs female GPs on the same register [22]. In India, more than 58% health workers are male, and around 25.7% women do not work in the workforce despite possessing a medical graduate degree. All this despite several government initiatives in recent years, including enhanced retirement age and suitable working conditions for female workers, to mainstream such technically qualified persons [23]. As compared to these countries, South Africa in terms of its male:female doctor ratio fares relatively well, however there is a continued need to understand HRH equity with a gender and racialized lens.

Gender equality in HRH has been recognized as a catalyst for all the Sustainable Development Goals (SDGs), alongside assertions of the invaluable role women can play as health-care providers [24]. However, it has been recognized that there exists a considerable gender disparity within the profession [25], as well as within global health leadership [26] that globally contributes to negatively affecting the health outcomes for women and children [27].

In this study, when forecasting for male doctors the workforce needed has been estimated at status quo, i.e. 4.69 per 10,000 population for male doctors and 3.21 for female doctors—which is subsequently scaled up to 4.69 for female doctors as well to strike the equity target. We did not use any other benchmarks here as we undertook a gender equity-based HRH forecasting exercise.

The WHO has identified a threshold on the need for health workers in the context of the Millennium Development Goals; and estimated that 2.28 skilled health professionals (midwives, nurses, and physicians) per thousand population were generally necessary to achieve 80% coverage of skilled birth attendance [28, 29]. This narrowly defined threshold became widely used to assess the adequacy of the supply of health workers around the globe. Also, WHO's "Health workforce requirements for universal health coverage and the Sustainable Development Goals" quantify through an innovative empirical approach, the health workforce requirements for the attainment of SDG 3. It suggests a new benchmark of 4.45 physicians, nurses, and midwives per thousand population [29]. Finally, the workforce SDG 3c talks about 40% of Member States not achieving a minimum of 1 medical doctor per 1000 [30]. So contextually, the norm of 1 medical doctor per 1000 population can be said to availability of a minimum and not a recommendation. However, in this HRH forecasting exercise we are not aiming for the 1 medical doctor per 1000 norm but consider the required supply to reach gender equity through workforce forecasting—targeting equal representation of women and men in the medical doctor workforce. In the current scenario if both genders are combined then doctor per 1000 population density will be 0.79 per 1000 (i.e. 0.47 male + 0.32 female). If we try to reach the 1:1 equity ratio for both genders then the final doctor:1000 population density will be 0.94 per 1000 population (0.47 male + 0.47 female), which indirectly would lead us to the overall course correction of HRH density (gold standard of 1:1000) while keeping in view the issue of gender inequity.

It seems governmental efforts to encourage women of colour to come forward and enter into the medical profession is bearing fruit, though such efforts need to be accelerated and sustained in future as well. With higher growth in the production of female doctors suggests that over time, it is possible to reach equitable representation in the medical workforce. Clear projections and estimations of the supply-side planning that is required to reach these targets within a timeframe have been absent from policy discussion for some time.

### Limitations

The HPCSA has data that describe who is registered, licensed, and accredited to practise in South Africa. The data do not indicate if practitioners live in South Africa, whether they are currently practising, either in a public or private sector or if they operate in a part-time or full-time capacity and thus should be viewed as the upper limit of supply. In order to explain the factors driving the quantitative trends, additional qualitative data needs to be adopted as a means to uncover the extent of the structural inequalities. A deeper sociological understanding of the effect of gender within these systems remains important to address these inequities in any meaningful way [17].

## Conclusion

The increasing feminization of the medical profession is a well acknowledged trend globally. However, as has been shown, this does not always lead to equitable representation of women within medicine, in terms of socio-demographic indicators (for example, population group and class), and/or across different specialties, leadership positions within the profession, regions and sectors of practice. An important pre-requisite for effective policy intervention (whether demand- or supply-side) is a baseline of understanding on the extent of disparity in female representation in medicine. This study provides a framework particularly for other African countries as well as lower and middle-income countries for the analysis of current availability and estimation of optimum HRH levels towards alternatively reaching gender equity targets.

## Data Availability

The datasets generated and/or analysed during the current study are not publicly available.

## Appendix: Medical specialists in South Africa (gender-wise break-up)

**Table:**

Medical specialists	Male	Female	Grand total	Male:female specialist
Anesthesiology	1216	638	1854	1:0.5
Cardiology	1	0	1	1:0
Cardiothoracic Surgery	139	9	148	1:0.1
Clinical Pharmacology	9	11	20	1:1.2
Community Health	47	30	77	1:0.6
Dermatology	139	129	268	1:0.9
Diagnostic Radiology	693	314	1007	1:0.5
Education Pathology (Microbiological)	1	0	1	1:0
Emergency Medicine	83	51	134	1:0.6
Family Medicine	714	255	969	1:0.4
Fast Track—Pediatrics	1		1	1:0
Medical Genetics	1	10	11	1:10
Medicine	1340	479	1819	1:0.4
Neurology	110	81	191	1:0.7
Neurosurgery	217	11	228	1:0.1
Nuclear Medicine	41	41	82	1:1
Obstetrics and Gynecology	875	436	1311	1:0.5
Occupational Medicine	21	16	37	1:0.8
Ophthalmology	433	127	560	1:0.3
Orthopaedics	910	48	958	1:0.1
Otorhinolaryngology	318	49	367	1:0.2
Paediatric	628	761	1389	1:1.2
Paediatric Surgery	17	17	34	1:1
Pathology	506	486	992	1:1
Physical Medicine (closed)	2	5	7	1:2.5
Plastic and Reconstructive Surgery	196	34	230	1:0.2
Preventive Medicine (closed)	10	2	12	1:0.2
Psychiatry	440	476	916	1:1.1
Public Health Medicine	19	54	73	1:2.8
Radiation Oncology	121	118	239	1:1
Radiology	0	1	1	1:0
Surgery	922	144	1066	1:0.2
Urology	284	15	299	1:0.1
Venereology (closed)	2	0	2	1:0

## Abbreviations

HRH: Human resources for health
HPCSA: Health Professions Council of South Africa
SA: South Africa
USA: United States of America
DENOSA: Democratic Nursing Organisation of South Africa
SANC: South African Nursing Council
HIV/AIDS: Human Immunodeficiency Virus/Acquired Immuno Deficiency Syndrome
TB: Tuberculosis
WHO: World Health Organization

## Footnotes

1 As noted in the introduction to the African National Congress’ (ANC) 1994 National Health Plan (ANC, 1994): “the South African government, through its apartheid policies, developed a health-care system which was sustained through the years by the promulgation of racist legislation and the creation of institutions such as political and statutory bodies for the control of the health-care professions and facilities. These institutions and facilities were built and managed with the specific aim of sustaining racial segregation and discrimination in health care”.

2 The paper uses both the terms gender equity and equality as both are relevant to the discussion. Gender equity refers to “fairness of treatment for women and men, according to their respective needs. This may include equal treatment or treatment that is different but which is considered equivalent in terms of rights, benefits, obligations and opportunities”, while gender equality would refer to equal outcomes for men and women.

3 In this paper, we have used the term population along the lines of the Population Registration Act (Act No. 30 of 1950) which classified South African citizens into four major population groups namely ‘White’, ‘Coloured’ ‘Indian’, ‘Chinese’ and ‘Black’ based on the colour of their skin (14). Although the legislation on race was repealed in 1991, in some instances it is still required to report along these categories in different sectors across government.

4 The limitation of this approach is the difficulties in an exact quantification of the attrition of medical doctors between graduation and practice.

5 Intersectionality suggests that various socially and culturally constructed categories of discrimination interact on multiple and often simultaneous levels, contributing to varying degrees of social inequality. For example, although women as a group may be discriminated against and have less power than men within a specific context, within a group of women, Black women are likely to be more disadvantaged and have less power within that context.
